# Impact of China's Vaccine Incidents on the Operational Efficiency of Biopharmaceutical Companies

**DOI:** 10.3389/fpubh.2020.00093

**Published:** 2020-04-08

**Authors:** Kuang-Cheng Chai, Ran Tao, Ke-Chiun Chang, Yang Yang

**Affiliations:** ^1^Business School, Guilin University of Electronic Technology, Guilin, China; ^2^School of Economic and Management, Wuhan University, Wuhan, China

**Keywords:** rate of return on total assets, social responsibility, operational efficiency, biopharmaceuticals, medical health

## Abstract

**Objectives:** This study aims at (1) analyzing the 15 representative biopharmaceutical companies in China before and after the vaccine incidents during the transition period of China's economy, using financial indicators as a proxy for corporate operational efficiency and corporate social responsibility (CSR); (2) analyzing the factors influencing business operational efficiency, and providing a future development strategy for the pharmaceutical industry to better fulfill its social responsibility by studying the relationship between business operational efficiency and social responsibility performance; and (3) conducting a comprehensive analysis of the results of this study, and providing relevant recommendations for biopharmaceutical companies to fulfill their social responsibilities and ensure a high quality of products.

**Methods and Data:** The panel data regression evaluates whether a biopharmaceutical company's social responsibility has an impact on its operational efficiency. A part of the data used in this study is obtained from the quarterly and annual financial statements of biopharmaceutical companies, while other relevant data are obtained from the China Stock Market & Accounting Research (CSMAR) database.

**Results:** A comprehensive regression analysis of the rate of return on total assets shows that the vaccine incidents have made biopharmaceutical companies pay more attention to CSR, as actively taking social responsibility will help establish a better corporate image and form, and increase the operating efficiency.

**Conclusion:** According to the current state of development in China, the size of a company has a positive impact on the improvement of its social responsibility and operational efficiency. The larger the company, the more attention it pays to shareholders and consumers, and the stronger its sense of responsibility to employees and society. By actively undertaking CSR activities, a company will get more support from shareholders, employees, and consumers, and will also enhance the corporate reputation. Biopharmaceutical companies actively undertake social responsibilities toward the government, employees, and shareholders, which will, to some extent, increase the public's trust in drug safety and have different degrees of impact on the overall efficiency of the company.

## Introduction

### Research Background

In recent years, China's economy has been developing vigorously with a steady growth, the national gross domestic product (GDP) has remained at a high level, and the increase in the people's disposable income has made the public pay more attention to the safety of people's livelihood in terms of medical care and drug supervision. Simultaneously, to improve the population development strategy, China has implemented a family planning policy. Hospitals across the country experience a high demand for vaccination of newborns. In general, China's demand for medical drugs is relatively high at this stage, and the safety of medical drugs is high, with the standards being even higher. However, there are also hidden risks. The vaccine incidents that have occurred since 2013 have not only brought about a crisis of public trust in biopharmaceutical companies, but also a powerful motivation for biopharmaceutical companies to improve their self-discipline and strengthen their internal social responsibility supervision. In 2018, a new round of vaccine incidents caused domestic biopharmaceutical vaccine companies to lose the public trust, thus inspecting the role of Chinese pharmaceutical companies and discussing whether they actively fulfill their social responsibilities, regulate internal production, and protect the people's livelihood.

Corporate social responsibility (CSR) means that while creating corporate profits and taking legal responsibilities toward shareholders and employees, companies consciously emphasize the value of people, society, environment, and consumers. In other words, it means assuming responsibility for the people, environment, and society, beyond the traditional corporate philosophy of making profits the sole goal. Biopharmaceutical companies play an important role in securing people's livelihood, since all families need medical drugs. Thus, biopharmaceutical companies should adopt CSR policies, establish a good corporate image, and ensure the safety of people's livelihood in China. Since China's economy is in a transition period with a steady growth, and owing to the nature of the market economy, biopharmaceutical companies have special identities. However, while pursuing economic benefits, they have to ensure the safety of drugs and protect consumers, as medical services are strongly related to the people's livelihood and the healthy and orderly development of the entire society, with the key feature of medicines laying in their quality, which should be reflected in the special social responsibilities the producing company bears. As a benchmark for the safety of the people's livelihood, biopharmaceutical companies should take the lead and fulfill their social responsibilities.

This study selects 15 industry-leading biopharmaceutical companies in China, including traditional Chinese medicine pharmaceutical companies, emerging biotechnology pharmaceutical companies, and biopharmaceutical companies with specialized technologies. By choosing a diverse sample and collecting more different types of data, the research results could be more general and thus make the conclusions more in line with the real-world situation of the different types of biopharmaceutical companies in China. It collects panel data from 15 biopharmaceutical companies, collates financial data for each quarter from 2010 to 2018, and conducts panel data regression to analyze the information. Additionally, it identifies the factors influencing enterprise operational efficiency and investigates the positive impact of social responsibility fulfillment by the listed companies on enterprises in the biopharmaceutical industry. Moreover, some CSR performance indicators can promote biopharmaceutical companies to better fulfill their social responsibilities and provide momentum for future development. Based on the results of this paper, combined with the current domestic economic transformation plan and the existing internal regulation mechanism of biopharmaceutical enterprises, we put forward suggestions for biopharmaceutical companies to fulfill their social responsibilities and ensure the high quality of their products.

## Literature Review and Hypothesis Development

### Literature Review

In view of the existing literature, although there are few domestic and foreign studies on the status of the biopharmaceutical industry listed companies in terms of their CSR performance, the related research focus not only is limited to CSR performance and policies but also investigates its positive influence on various aspects of the companies' management. CSR, as a positive revision of the company's decision to make profits at the present stage, does not take profit as the starting point, and assumes more responsibilities toward the government and society, which is conducive to gaining the public trust by biopharmaceutical enterprises. Indeed, an increasing number of scholars have proposed that the realization of corporate profit objectives is not inconsistent with respecting the interests of other stakeholders.

In the literature on the relationship between corporate research and development (R&D) investment and business efficiency, Albareda et al. (1) analyzed the CSR public policies of developed European democracies; in particular, they chose 15 EU countries, and constructed the “Partnership, community business, sustainability, citizenship and assembly” models, incorporating CSR public policies into a broader approach to social governance. In recent years, Omar and Zallom (2) used the Tobin's *Q* ratio as a dependent variable to measure the market value of a company. Their regression analysis included size and leverage, which may have an impact on the relationship between CSR and a company's market value, with the results showing that investment in the field of CSR may have a negative or no impact on market value. Barić (3) studied the impact of corporate social value on stakeholders and proposed that when a company assumes social responsibility and contributes to the society, all stakeholders can continue to provide motivation and support for the corporate development to achieve mutual promotion and coordinated development of the community of interests. Garde Sánchez et al. (4) demonstrated the impact of CSR information disclosure on state-owned enterprises. The results showed that the size of a state-owned enterprise, the CSR of an industry, and managers are the most important factors affecting the online disclosure of CSR information. van Doorn et al. (5) suggested that although many studies have reported the positive impact of CSR on the customer attitude, the effectiveness of CSR initiatives varies widely among consumers, brands, and companies. A study of 1375 customer feedbacks from 93 brands in 18 industries confirmed that adopting CSR policies would indeed result in a more positive customer attitude and a higher customer retention rate. Fanti and Buccella (6) analyzed the duopoly market equilibrium of CSR behavior under management commission, with the results showing that under the equilibrium state, for socially responsible enterprises, the goals of profitability, consumer satisfaction, and social welfare are all achieved. Abeysekera and Fernando (7) used environmental performance as a representative of CSR, and found that family businesses have greater responsibility to shareholders than non-family businesses, as they must take care of all shareholders due to the lack of diversified business models of controlling families, with financial interests causing a better CSR fulfillment. Frederiksen (8) studied how metal mining companies understand and implement CSR activities and analyzed why CSR projects are considered strategic challenges for managing enterprises, showing that CSR has become the focus of risk management. Gandhi and Raina (9) found that social entrepreneurship is gradually becoming an important factor in global discussions on volunteerism and civic commitment, with social entrepreneurs emphasizing how to reduce or eliminate social pressure, and non-social entrepreneur types having a more important position. Moratis and van Egmond (10) examined the relationship between the CSR performance level, the practice degree of emerging market companies, and industry effects, showing that companies may strategically use CSR to compensate emerging markets or divert the stakeholders' attention to emerging markets.

The research on CSR in China has focused more on the people's livelihood such as the food and drug industries. The active and independent implementation of social responsibility by relevant enterprises in these industries is of great significance to the long-term development of enterprises. Na and Jian (11) constructed a conceptual model of the relationship between CSR, brand equity, consumers' emotions, and rational cognition in their research. Simultaneously, the structural equation method was used to test the relationship between CSR and brand equity dimensions, and the mediation effect. The results showed that corporate environmental responsibility directly affects brand equity, and consumer responsibility directly or indirectly affects brand equity through the perceived quality. Xie et al. (12) constructed an evolutionary game model for enterprises and the public under government intervention and explored the strategic choices for enterprises with respect to undertaking social responsibility or public behavior supervision. The results showed that when government incentives are strong enough, companies with stronger brand awareness are more willing to fulfill their social responsibilities. Peng et al. (13) used the hierarchical linear model to test whether corporate R&D innovation and its synergies affect corporate value. Simultaneously, taking corporate size as a reference, they found that the synergies of R&D innovation cannot have a positive impact on the shareholder value. Han (14) used a sample of 29 Chinese smart-city companies from 2011 to 215, to study the effect of heterogeneous thresholds of' independent R&D on financial performance, showing that different threshold constraint conditions causes differences in enterprise distribution, with the influence of the independent R&D of smart-city enterprises on financial performance varying according to factors such as capital structure and equity concentration In addition, independent R&D negatively affect the enterprise operational efficiency when the concentration of enterprises is high. Ge and Zhao (15) investigated the impact of corporate social media capabilities on the relationship between CSR characteristics and corporate double reputation. The results showed that CSR can have a positive effect on the reputation of a business.

In summary, the CSR literature is abundant, with the conclusion being that enterprises should assume relevant responsibilities toward the government, employees, creditors, resources, and environment, and not only set their goals to improve economic benefits. Although the domestic research on CSR, compared to the international literature, is relatively underdeveloped, from the innovation perspective, domestic enterprises focus on CSR in industries related to the people's livelihood, which have an enormous market demand, as in the case of biopharmaceutical enterprises. In combination with the vaccine incidents in recent years, biopharmaceutical enterprises experience high demand; however, owing to the lack of corresponding supervision and management, quality and safety problems occur from time to time, which may affect the public's trust in these enterprises, thus verifying the importance of CSR for the improvement of operational efficiency. In the literature on the social responsibilities assumed by enterprises at home and abroad, there is no consensus on the effects of CSR practices on the long-term operational efficiency, as some scholars argue that CSR can have a positive impact on management efficiency, whereas others are skeptical and point out that in the short term it might cause a decline in the efficiency of enterprise management.

Based on the previous literature review, enterprise stakeholders can obtain a more comprehensive evaluation of enterprises through the CSR reports of non-financial information, that is, the CSR report will help enterprise creditors, governments, employees, shareholders, and other stakeholders to fully understand the social responsibility fulfillment and business situations of enterprises, so that they can better reduce the information asymmetry, improve operational efficiency. Therefore, this paper proposes a research hypothesis: listed biopharmaceutical enterprises can improve their operational efficiency by fulfilling their social responsibilities.

### Liability to Creditors and Operational Efficiency

The purpose of an enterprise is to make profits; however, China's corporate law clearly stipulates that the purpose of a company includes protecting the legitimate rights and interests of shareholders and creditors. Therefore, it can be inferred that it is also an important social responsibility for an enterprise to strengthen the protection of creditors. Dam and Scholtens (16) linked CSR with key financial accounting ratios in their research, and concluded that there is a positive relationship between CSR and financial performance, although this relationship is based on the condition of financial performance indicators. Enterprises must analyze the particularity of creditors and find more effective ways to protect their rights and interests to achieve long-term development of the company. Moreover, this study uses earnings per share (EPS) to represent the social responsibility of biopharmaceutical companies due to creditors, with the higher the EPS, the stronger the biopharmaceutical company's financial profitability, the more it can gain the trust of current and potential investors and improve the company. Therefore, this study proposes the following hypothesis:

H1: The social responsibility performance of biopharmaceutical enterprises with respect to creditors has a relevant impact on the operational efficiency.

### Responsibility Toward Government and Operational Efficiency

It is particularly important for biopharmaceutical enterprises to fulfill their social responsibilities toward the government due to the particularity of the industry. Velte and Stawinoga (17) conducted research on 53 CSR guarantees, finding that corporate internal governance may be a factor affecting CSR decisions. In addition, after a detailed analysis of the market level, they found that companies if want to achieve mutual benefits, they must strengthen their internal management and establish good relations with the government. This study uses the contribution rate of the government, which is the ratio of the various taxes paid minus the tax refunds received to the main business income. Additionally, the social responsibility of biopharmaceutical enterprises toward the government is of utmost importance, since the government's main finance source is the tax revenue, while the governmental contribution to enterprises boosts their income, with the higher the ratio, the better the CSR performance of biopharmaceutical companies. Therefore, we put forward the next hypothesis:

H2: The social responsibility performance of biopharmaceutical enterprises with respect to governments has a relevant impact on their operational efficiency.

### Responsibility Toward Employees and Operational Efficiency

The social responsibility of enterprises toward employees is to safeguard the rights and interests of employees and to provide various benefits that employees should enjoy. Wang and Zhang (18), based on a Chinese government report, improved the negotiation and coordination mechanism involving the government and enterprises. They suggested that stable labor relations can promote the healthy development of enterprises, and that establishing harmonious labor relations is the social responsibility of enterprises. As the internal stakeholders of an enterprise, major enterprises should pay more attention to their rights and interests to protect their employees. In this study, the contribution rate of employees is taken as a measure of the CSR to employees. The higher the contribution rate of employees, the more importance the enterprises attach to the development of employees, thus actively assuming the social responsibility toward employees and improving the operational efficiency of enterprises. Therefore, we propose the following hypothesis:

H3: The social responsibility performance of biopharmaceutical enterprises has a relevant impact on their employees and the operational efficiency of the enterprises.

### Responsibility Toward Shareholders and Operational Efficiency

The primary goal of an enterprise is to maximize its interests and the shareholders' benefits. The shareholders, as investors, are supposed to fulfill their social responsibilities, and the enterprise shall be responsible for the security of the shareholders' funds and eliminate any arbitrary waste of them. Simultaneously, the enterprise shall regularly report to the shareholders information on major issues and decisions, financial statements, and other information, and ensure the authenticity of information. We use the return on equity (ROE) to describe the social responsibility of biopharmaceutical enterprises toward shareholders. Therefore, we present the following hypothesis:

H4: The social responsibility performance of biopharmaceutical companies with respect to shareholders has a relevant impact on their operational efficiency.

### Other Relevant Parts and Operational Efficiency

The debt asset ratio (DAR) of a biopharmaceutical enterprise is an index that measures its ability to use the funds provided by creditors to carry out business activities. It reflects the proportion of funds provided by creditors to all funds and the degree of protection of enterprise assets in terms of the creditors' rights and interests. Meanwhile, the total assets (TA) of a biopharmaceutical enterprise reflect the scale of the enterprise to some extent and its overall profitability. Therefore, we propose the following hypotheses:

H5: The DAR of a biopharmaceutical enterprise has a relevant impact on its operational efficiency.
H6: The scale of a biopharmaceutical enterprise has a relevant impact on its operational efficiency.

## Methodology and Measurement

### Sample and Data Collection

This study selects Shanghai and Shenzhen A-share listed biopharmaceutical companies from 2010 to 2018 as samples. The financial data for each quarter of the period are collected and used to study the impact of Chinese biopharmaceutical companies' CSR performance on their operational efficiency during the country's social and economic transition. Among the data used in this study, some data are collated from the annual financial statements of biopharmaceutical enterprises, while other relevant data are all obtained from the China Stock Market & Accounting Research (CSMAR) database.

The research samples are selected according to the following conditions: (1) non-biopharmaceutical enterprises and enterprises listed before 2010 are excluded, since the research objects of this study only include biopharmaceutical enterprises; (2) the biopharmaceutical enterprises with missing or abnormal data are eliminated, since the missing parts will reduce the sample size and affect the sample integrity, thus affecting the data modeling and analysis, and consequently, to some extent, the results; (3) the enterprises with a higher size than the general level are excluded, since the excessive TA of some biopharmaceutical enterprises will lead to a large difference between them and other samples, causing the data modeling to be too large, which may affect the universality of the analysis results, making the results unrepresentative. Finally, 15 biopharmaceutical companies, including traditional Chinese medicine pharmaceutical companies, emerging biotechnology pharmaceutical companies, and biopharmaceutical companies with special technologies in this study, were obtained.

### Variables' Definitions and the Measurement Model

#### Variables' Definitions

The definitions of related variables are presented in Table 1. The rate of return on total assets (*RRTA*) is the enterprise net profit and total assets ratio of the total amount on average, which, as an indicator, reflects the effect of comprehensive utilization of the enterprise assets. Additionally, it is a measure of enterprises that uses the total amount of creditors and owners' equity, as one of the important indices for profit. It can measure the enterprise operational ability, comprehensive consideration, and operational efficiency. *EPS* refers to the after-tax profits and total equity ratio, as each common shareholder has to bear his/her part of the enterprise's net profit or net loss, reflecting the operating results of the enterprise. Thus, it measures the common profit level and investment risk, and can be used to evaluate the enterprise profit ability, predict its growth potential, and manifest the enterprise's social responsibility toward the creditors. The government contribution rate (*GCR*) is the ratio of the various taxes paid minus the tax refunds received to the main business income, which reflects the obligation of biopharmaceutical enterprises to pay tax voluntarily and reflects their social responsibility to the government whose main finance source is the tax revenue. The employee contribution rate (*ECR*) is the ratio of the cash paid to and for employees to the income from main business, which, to some extent, reflects the salary and welfare treatment of the employees of biopharmaceutical enterprises and the social responsibility of biopharmaceutical enterprises to employees. *ROE* is a financial indicator that reflects the income level of shareholders' equity. It can measure the utilization efficiency of the capital invested by an enterprise for the shareholders, reflect the profitability of the enterprise, and evaluate the social responsibility of biopharmaceutical enterprises to shareholders.

**Table 1:** Definitions of variables.

**Variables**	**Symbol**	**Definition**
Rate of return on total assets	*RRTA*	Total net profit/Average share of total assets × 100%
Earnings per share	*EPS*	(Current gross profit—preferred stock dividends)/total share capital at the end of the period
Government contribution rate	*GCR*	(Payments of taxes and fees—refund of taxes and fees received)/Main Business Income × 100%
Employee contribution rate	*ECR*	Cash and Main Business Income Payments to Employees and Employees × 100%
Return on equity	*ROE*	Return on equity = After-tax profit/Owner's equity
Debt asset ratio	*DAR*	Debt Asset Ratio = Total liabilities/Total assets × 100%
Total assets	*TA*	All assets owned or controlled by commercial banks that bring economic benefits

The control variables are *DAR* and *TA*, in which the natural logarithm of the total assets is taken. The *DAR* is the ratio of total debt to *TA*, which measures how biopharmaceutical companies use the creditors' ability to provide capital for business activities. In other words, the *DAR* reflects the proportion of total capital provided by the creditors and the degree to which the creditors' rights and interests guarantee the enterprise assets, thus being, in biopharmaceutical enterprises, a comprehensive evaluation indicator of anti-risk ability. *TA* is used to reflect the scale of biopharmaceutical companies, encompassing all what they own or control and can bring economic benefits. The overall profitability of biopharmaceutical companies is the natural logarithm of *TA*. To make the calculation more convenient, reduce the data of absolute value, smooth the data, abate the collinearity of the model, and tackle the problem of heteroskedasticity, we construct a major evaluation index of biopharmaceutical enterprise on an industrial production scale.

#### Regression Model

This study establishes the following regression model:

(1) $$\begin{array}{lll} Y_{it} & = & \beta_{0}+\beta_{1}EPS_{it}+\beta_{2}GCR_{it}+\beta_{3}ECR_{it}+\beta_{4}ROE_{it}\\ \;+ & \beta_{5}DAR_{it}+\beta_{6}TA_{it}TA+\varepsilon_{it} \end{array}$$

In the model, *Y* is the *RRTA* of biopharmaceutical enterprises, which represents the operational capacity of biopharmaceutical enterprises, *i* represents a firm, and *t* represents time.

## Empirical Analysis

### Descriptive Statistics and Correlation Analysis

The descriptive statistics for the variables introduced into our model are shown in Table 2. Among them, the minimum and maximum values of *RRTA* are 0.2115 and 0.2513, respectively, indicating that the outliers have been eliminated. The biopharmaceutical companies selected as the source of the sample have comparable operating conditions, with an average value of 0.2342, indicating that the average profit of a biopharmaceutical company is about 23%. The variance of each group of data selected as the explanatory variable is <0.5, the variance of the control variable is also <0.6, and the degree of data dispersion is generally small. In summary, the range of data selected is relatively comprehensive and reasonable.

**Table 2:** Descriptive statistics.

	**Min**	**Max**	**Mean**	**SD**
*RRTA*	0.212	0.251	0.234	0.007
*EPS*	−2.180	3.340	0.556	0.629
*GCR*	0.006	0.276	0.098	0.046
*ECR*	0.006	0.219	0.104	0.051
*ROE*	−5.008	0.289	0.049	0.319
*DAR*	0.076	0.991	0.445	0.171
*TA*	21.149	25.127	23.424	0.730

The correlations matrix of this study is shown in Table 3, which shows *RRTA, EPS, GCR, ECR*, and *ROE*. In addition, for the control variables, it shows a positive correlation between *TA* and the control variable, a negative correlation between *DAR* and *ECR*, and that the correlations of *TA* are not strong, with all relationships being characterized by high correlation.

**Table 3:** Correlation matrix.

	***RRTA***	***EPS***	***GCR***	***ECR***	***ROE***	***DAR***
*RRTA*	1					
*EPS*	0.617^**^	1				
*GCR*	0.548^**^	0.200^**^	1			
*ECR*	0.181^*^	−0.023	0.565^**^	1		
*ROE*	0.720^**^	0.647^**^	0.338^**^	0.114	1	
*DAR*	−0.662^**^	−0.385^**^	−0.614^**^	−0.298^**^	−0.432^**^	1
*TA*	0.015	0.182*	0.026	−0.042	0.012	−0.003

** p < 0.01,

* *p < 0.05*.

To further test the hypotheses and confirm the preliminary inference, the table of significance correlation coefficients is used for analysis, as shown in Table 3. Table 3 shows the correlations between the *RRTA* and *EPS, GCR, ROE*, and *DAR* have passed the 1% significance test, showing a significant correlation. The correlation between the *RRTA* and the *ECR* has passed the 5% significance test, showing a significant correlation. The correlation between the *RRTA* and *TA* has not passed the significance test; thus, there is no significant correlation.

From the regression results shown in Table 4, *EPS* shows a significant positive correlation with *RRTA*, with the *p* value being 0.005. Using the 1% significance test, the *EPS* and *RRTA* of biopharmaceutical companies are highly positive correlated, confirming that the biopharmaceutical enterprises have to actively bear the social responsibility toward creditors to improve their management efficiency.

**Table 4:** Results of regression model.

**Variables**	***RRTA***	***t* value**	***p* value**
*Cons*	0.170	1.354	0.179
*EPS*	0.017	2.881	0.005^**^
*GCR*	0.398	3.132	0.002^**^
*ECR*	−0.122	−1.307	0.194
*ROE*	0.121	5.155	0.000^**^
*DAR*	−0.117	−3.936	0.000^**^
*TA*	−0.004	−0.721	0.473
*F* value		41.780^**^	
Adj-*R*^2^		0.702	

** *p < 0.01*.

The *GCR* and explained variables show significant positive correlation with the *RRTA*, with the *p* value being 0.002. Using the 1% significance test, there is a highly positive correlation between *GCR* and *RRTA* in biopharmaceutical enterprises, proving that these enterprises have to actively assume social responsibility toward the government to improve its management efficiency.

*ECR* and *RRTA* have a negative correlation, with the *p* value being 0.194, thus not passing the significance test, and showing that the issuance of employee benefits in biopharmaceutical companies does not necessarily lead to the decrease of *RRTA*; that is, the management efficiency of biopharmaceutical enterprise management may not be affected by the evident.

The *ROE* and the explanatory variables show significant positive correlation with *RRTA*, with the *p* value being 0.000. Using the 1% significance test, the yield of biopharmaceutical enterprises' net assets and *RRTA* are highly positively correlated, proving that biological pharmaceutical enterprises have to actively undertake social responsibility toward shareholders to improve its management efficiency.

We can reasonably conclude that there is no significant correlation between *ECR* and *TA* and *RRTA* of biopharmaceutical enterprises. The reasons are as follows:

During the early stage of the adjustment of China's food and drug regulatory laws and regulations from 2010 to 2018, the relevant government control system has not improved, and the calculation of some indicators of various biopharmaceutical enterprises has not been agreed upon, which may lead to some errors in the data itself, thus affecting the hypotheses' results.

The sample size selected is not wide enough, as it consists of only 15 biopharmaceutical companies, including some traditional Chinese medicine pharmaceutical companies, emerging biotechnology pharmaceutical companies, and biopharmaceutical companies with special technologies. The sample is not sufficient and has limitations. The sample size and related data are not complete enough to affect the results of the hypothesis. As can be seen from Table 4, Adj-*R*^2^ is 0.702, verifying that the model has a good fit, and indicating that the explanatory variables have a strong ability to clarify the explained variables, with the effect of control variables being relatively obvious. Therefore, it is reasonable to choose relevant variables for the regression analysis, for the results to have a high credibility.

The comprehensive regression analysis of *RRTA* shows that the occurrence of the vaccine incidents has made biopharmaceutical companies pay more attention to CSR activities. Additionally, actively taking social responsibility will help establish a better corporate image and form. According to the current state of development in China, the size of a company has a positive impact on the improvement of social responsibility and operational efficiency. The larger the company, the more it pays attention to shareholders and consumers, the stronger its sense of responsibility to employees and the society, and the more active it adopts CSR policies. Thus, it will get more support from shareholders, employees, and consumers, and will also have a stronger corporate reputation.

Although results of the data analysis show that the increase in the *TA* of a biopharmaceutical company may not lead to a decrease in the turnover rate of *TA* and that the operational efficiency of the biopharmaceutical company may not change at this time, the expansion of the enterprise scale is conducive to a better fulfillment of social responsibilities, resulting in an increase in the operating efficiency of the company. Simultaneously, the biopharmaceutical companies' commitment to social responsibility is improving, as they pay more attention to internal supervision and actively assume social responsibilities toward governments, employees, and shareholders, thus increasing the public's trust in drug safety to some extent, which has different degrees of impact on the overall operational efficiency of the enterprise.

## Discussion and Conclusions

This paper selects 15 Chinese biopharmaceutical companies listed on Shanghai and Shenzhen stock markets as the research object, including some traditional Chinese medicine pharmaceutical companies, emerging biotechnology pharmaceutical companies, and biopharmaceutical companies with special technologies. The regression analysis on the quarterly financial data of various representative indicators and the financial measures evaluating the operational efficiency of biopharmaceutical companies in China facing the vaccine incidents during the country's economic transition show that a better performance with respect to fulfilling social responsibilities will have an impact on its operational efficiency of a country. Simultaneously, by referring to the macroeconomic data of the biopharmaceutical industry obtained from 2010 to 2018, the industry development situation can be derived.

There is a high positive correlation between the *EPS* and *RRTA* of biopharmaceutical companies. Thus, biopharmaceutical enterprises should actively assume social responsibility to creditors to improve their operational efficiency.

There is a high positive correlation between the *GCR* and *RRTA* of biopharmaceutical enterprises. Thus, biopharmaceutical enterprises have to actively assume social responsibility toward the government to improve their operational efficiency.

There is a high positive correlation between the *ROE* and *RRTA* of biopharmaceutical enterprises. Hence, biopharmaceutical enterprises need to actively assume social responsibility to shareholders, which is conducive to improving their operational efficiency.

There is a high positive correlation between the *DAR* and *RRTA* of biopharmaceutical companies. The increase in the *DAR* of biopharmaceutical enterprises will lead to an increase in the *RRTA*.

In general, China's biopharmaceutical companies are still underdeveloped with regard to fulfilling their social responsibilities. Although the social responsibility performance of creditors, the government, and shareholders is relatively in place, they do not show a high level of social responsibility toward employees and consumers. Biopharmaceutical companies, as people's livelihood enterprises with a more urgent national demand, must improve their product quality as soon as possible, ensure product safety, and better fulfill their social responsibilities. To ensure national safety, and improve the people conditions under the current development requirements of social and economic transformation, the level of public trust has to increase to meet the future development requirements of biopharmaceutical companies and achieve a healthy and sustainable growth.

At present, traditional biopharmaceutical enterprises in China still produce relatively high pollution emissions. A large amount of the chemical industrial waste is produced by the traditional production mode of the biopharmaceutical industry, which continues to consume resources and the environment to a high degree, for example, during the process of vaccine cultivation. The biopharmaceutical enterprises must increase investment in R&D to develop a new mode of production, improve the existing industry pattern, gradually make the transition to a new pharmaceutical industry, apply a variety of international emerging pharmaceutical production methods, improve cultivating modes to meet the needs of green transition and economic development, actively undertake the social responsibility toward the government, and improve the management efficiency.

Compared to small- and medium-sized biopharmaceutical enterprises, large biopharmaceutical enterprises in China are ahead of the time in terms of industrial chain upgrade, thus having a more perfect product quality and internal safety supervision. However, owing to the immature development mode, they are still limited at the present stage. Additionally, food and drug administration should be planned by the state to address drug safety problems facing biopharmaceutical enterprises of different sizes by imposing rectification requirements, adopting internal self-improvement regulations for the transformation and upgrading of enterprises, ensuring product quality and drug safety, reducing pollution, improving the management efficiency of management, and realizing the national green economic development goals.

With respect to the environment, since the development of the biopharmaceutical industry in China started relatively late, the production technology and equipment are underdeveloped compared to European countries, the United States, and other developed countries. Thus, the competitiveness of the Chinese domestic biopharmaceutical industry is weak, which makes some people more willing to seek imported special drugs overseas. However, traditional Chinese biopharmaceutical companies can utilize this opportunity by drawing on national capital, investing in R&D innovation and new technologies, improving their competitiveness, gaining a higher degree of public support, developing a green industry chain production mode, achieving industrial upgrading, and improving operational efficiency.

## Data Availability Statement

The datasets generated for this study are available on request to the corresponding author.

## Author Contributions

All authors designed and conducted the study, analyzed the data, wrote the draft, contributed to the interpretation of results, critically reviewed the draft, and approved the final manuscript.

### Conflict of Interest

The authors declare that the research was conducted in the absence of any commercial or financial relationships that could be construed as a potential conflict of interest.
